# Community-based intervention for management of diabetes in Nepal (COBIN-D trial): study protocol for a cluster-randomized controlled trial

**DOI:** 10.1186/s13063-018-2954-3

**Published:** 2018-10-22

**Authors:** Bishal Gyawali, Dinesh Neupane, Abhinav Vaidya, Annelli Sandbæk, Per Kallestrup

**Affiliations:** 1 Department of Public Health, Aarhus University, Aarhus, Denmark; 2 Nepal Development Society, Bharatpur, Nepal; 3 Department of Epidemiology Welch Center for Prevention, Epidemiology, and Clinical Research Johns Hopkins Bloomberg School of Public Health Baltimore, Maryland, Baltimore, USA; 4 Department of Community Medicine, Kathmandu Medical College, Kathmandu, Nepal

**Keywords:** Female community health volunteers, Primary healthcare, Non-communicable diseases, Type 2 diabetes, Community-based, Community health worker, Nepal

## Abstract

**Background:**

Type 2 diabetes is one of the fastest emerging chronic diseases in low- and middle-income countries. Population-based approaches, such as involvement of lay health workers offering culturally appropriate diabetes health promotion, may be the blueprint for the management of type 2 diabetes. This study aims to examine the effectiveness of a family-based home health education intervention on type 2 diabetes provided by female community health volunteers (FCHVs) in a semi-urban area of Lekhnath Municipality of Nepal.

**Methods:**

The COmmunity-Based INtervention for management of Diabetes in Nepal (COBIN-D) trial is a community-based, open-label, two-armed, cluster-randomized trial with seven randomly selected intervention and seven wait-list control clusters. A total of 112 subjects with type 2 diabetes will be recruited from the intervention clusters and 112 subjects from the wait-list control clusters. Based on the Health Belief Model and Social Support Theory, a 12-month family-based lifestyle intervention will be administered through FCHVs. Wait-list control clusters will continue to manage their glycemic condition as usual and their intervention will be delayed for 12 months. Participants will be measured at the beginning of the study and 12 months later. The primary outcome measure of the study will be difference in mean change (from baseline to 1 year) in fasting blood glucose between the two study arms. Impacts will be estimated using intention-to-treat analysis.

**Discussion:**

The COBIN-D is the first study investigating the effect of family-based home health education and screening on blood sugar levels in adults by FCHVs at community level in Nepal. The perspective of this study is to develop and implement, in collaboration with the community, a community-based, culturally sensitive diabetes prevention and control program. It is anticipated that the study can act as a feasible and affordable tool for evidence-based integrated care for improvement of diabetes management and outcomes in Nepal as well as in other low- and middle-income countries.

**Trial registration:**

ClinicalTrials.gov, Identifier: NCT03304158. Registered retrospectively on 03 October 2017.

**Electronic supplementary material:**

The online version of this article (10.1186/s13063-018-2954-3) contains supplementary material, which is available to authorized users.

## Background

While communicable diseases remain an important public health issue in low- and middle-income countries (LMICs), the rising burden of non-communicable diseases (NCDs) and their risk factors poses a double burden on health systems [1]. Diabetes is increasingly becoming a significant public health problem worldwide, leading to high morbidity and mortality resulting from clinically severe complications. Global estimates for 2014 suggest that the number of people with type 2 diabetes was 422 million (8.5% of the world’s adult population), with the number projected to increase to 642 million by 2035 and over 75% of new adult type 2 diabetes cases occurring in LMICs [2]. In particular, type 2 diabetes is becoming more widespread globally, accounting for over 90% of all diabetes cases [3]. The rising prevalence is attributed to various modifiable risk factors, such as changes in nutrition and lifestyles leading to physical inactivity and obesity, as well as non-modifiable risk factors including family history and age-related factors [4]. Furthermore, LMICs not only have higher predicted increases in prevalence rates, but people diagnosed with diabetes living in LMICs also have worse blood glucose control compared to those living in high-income countries [5].

Type 2 diabetes has reached epidemic proportions in the South-East Asia region, leading to significant increases in morbidity and mortality in recent years [6]. Indeed, in our previous systematic review and meta-analysis [7], we reported that type 2 diabetes is emerging as a major healthcare problem in Nepal, with a prevalence rate of 8.4%; nevertheless, as the review suggests, there is minimal data regarding diabetes prevalence and risk factors in Nepal [7]. Additionally, another study reported that more than 50% of people with diabetes in Nepal remain undiagnosed [8]. This is unsurprising since the prioritization of communicable and other infectious diseases has historically overshadowed NCDs, including diabetes, in Nepal [8]. Most health facilities in Nepal often lack NCD health staff; therefore, to address the current healthcare crisis, task-shifting among healthcare providers to allow mid-level cadres, such as lay health workers, to cope with the workload is urgently required. The country has made significant progress in reducing maternal mortality rates over recent years despite the low socioeconomic conditions, political instability, maldistribution of human resources for health, and lack of quality care in health facilities. Approximately 50,000 female community health volunteers (FCHVs) have been contributing to key public health programs in Nepal, including family planning, maternal and neonatal health, and child health, and are often credited for the improvement in maternal health in rural Nepal [9]. FCHVs are local women who act as lay health workers in their communities, with limited formal education, serving voluntarily within the government system in all the villages of Nepal; they are recruited from each Village Development Committee (‘village’), and trained to provide basic community health services [10]. FCHVs receive 18 days of basic training as well as periodic refresher and program-specific training to provide community health services. They form the basis of the Nepalese community-based primary healthcare system and act as a key referral link between the health service system and the community [11]. The implementation of the FCHV program in Nepal has been identified as a successful strategy to address health problems at the community level since the late 1980s [12]. However, there is no evidence regarding the involvement of FCHVs in the prevention and management of diabetes in the country. One possibility of FCHV involvement could be through interventions offering culturally appropriate diabetes health promotion, which could act as the blueprint for the management of the burgeoning prevalence of diabetes in Nepal.

A population-based approach to reduce the blood glucose levels by a small amount or to prevent the increase in blood glucose with age can lead to a marked reduction in the risk of type 2 diabetes and could prevent the progression of microvascular complications [13]. It is predicted that application of population-based interventions to target modifiable risk factors could prevent at least 80% of NCDs, including type 2 diabetes [14]. A review of the role of community health workers in improving diabetes outcomes reported that community health workers delivering a lifestyle intervention led to a significant effect on glycemic and fasting blood glucose levels at 6–12 months [15]. A more recent systematic review and meta-analysis of group-based diabetes self-management education similarly concluded that the intervention had as significant effect on glycemic and fasting blood glucose levels at 6–12 months [16]. However, none of the studies in this review had been conducted in LMICs and most of the studies involved highly trained staff such as doctors and specialist nurses or dieticians. One study on health promotion for blood glucose reduction in rural Africa yielded inconclusive results [17]. Some studies conducted in resource-poor settings exhibited limitations in reporting results, including lack of true randomization [18] or control groups [18, 19]. Therefore, there is still a lack of evidence on effective protocols for community health worker population approaches to reducing blood glucose in diabetic patients.

Herein, we describe the protocol of a study designed to address gaps in the management of diabetes by community health workers, called the Community-Based Intervention for management of Diabetes (COBIN-D). The COBIN-D is a cluster randomized controlled trial (cRCT) to explore the potential role of FCHVs in diabetes management at the community level in Nepal. The primary aim is to evaluate the effect of a family-based home health educational intervention administered by FCHVs on the reduction of blood glucose levels among diabetic individuals.

## Methods

This manuscript adheres to the Consolidated Standards of Reporting Trials (CONSORT) 2010 Statement extension for cRCTs [20].

### Trial design

The COBIN-D trial is a community-based, open label, two-arm cRCT with equal allocation of participants between intervention and wait-list control arms. The trial will be conducted for 12 months, including baseline and follow-up outcome assessments.

### Study setting

The study will be conducted in a semi-urban area of Lekhnath Municipality of Nepal. The study area is now named the Pokhara Metropolitan City due to recent restructuring of the state of Nepal according to the concept of a democratic federal system. However, our study will still be implemented in our earlier defined study area. According to the 2011 census, the Lekhnath Municipality has a total population of 58,816 in 14,937 households, and is administratively divided into 15 smaller units called clusters (wards). The municipality has a literacy rate of 85%, life expectancy of 59.7 years, and sex distribution of 31,951 females and 26,865 males [21]. According to the data on district health workers, there were 123 FCHVs in the municipality in 2013. No community-based interventions for diabetes at the population level have been carried out in this region to date. We aim to conduct our study in conjunction with the Community-Based Management of Hypertension in Nepal (COBIN) study [22], which is being carried out in the region as a 3-year PhD project from Aarhus University. The COBIN study is designed to explore the effectiveness of FCHV-led interventions in hypertension control at the community level. Since hypertension and diabetes share major risk factors, it is anticipated that combining a home health education program delivered by FCHVs for both diseases would help to effectively prevent the disease at an early stage. In addition, there is potential for great synergies when carrying out the study in collaboration with an already established project with a similar mission.

### Recruitment procedures

We adopted the sampling frame from the COBIN study [22], including a population framework of all eligible participants using the election voter’s list for 2007 (Lekhnath). The voter’s list contained information about the household. The COBIN study conducted a baseline survey using this voter list, and prepared a list of eligible respondents to participate in the trial. If there was more than one participant from the same household eligible to participate in the study at the time of data collection, the Kish method was adopted to select the participant [23].

### Participants

**Fig. 1:**
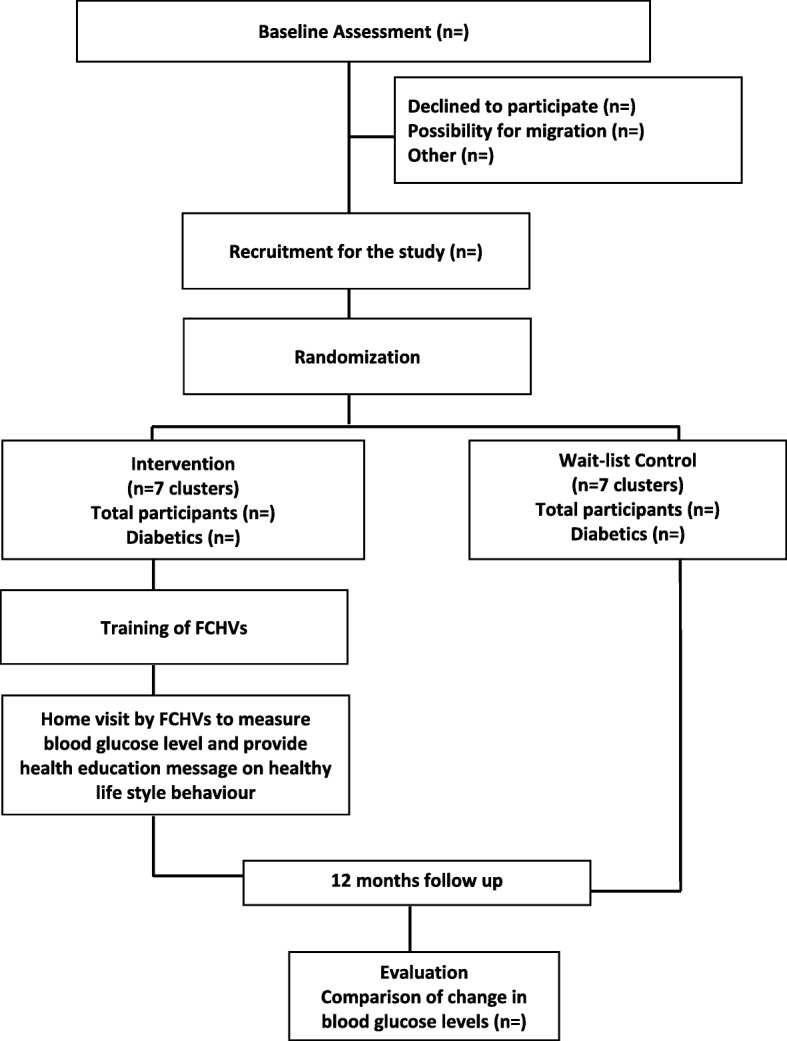
Planned flow of participants through the trial. *FCHV* female community health volunteer

The populations targeted in our study area are listed in relation to the COBIN study [22]. We will invite participants listed in the COBIN study for a baseline survey. At first, baseline data will be collected in each participating cluster. During the baseline survey, diabetics will be identified and recruited for the trial. The diagnosis will be made based on the 2006 World Health Organization (WHO) guidelines [24]. The flow of trial participants is shown in Fig. 1, which includes the number of participants screened, eligible, excluded, recruited, randomized, and analyzed for the primary outcome [22]. The SPIRIT checklist for this trial is provided as an Additional file 1.

### Clusters

Although the municipality is composed of 15 clusters, only 14 will be selected for the trial. We will exclude one cluster, as in the previous study [22], because it is different in terms of sociodemographic and geographical distribution. Allocation of the clusters into intervention and control arms is described in the section on randomization.

### Inclusion and exclusion criteria

Participants included in the 25–64 years age group in the COBIN study are eligible for inclusion in the baseline survey. Those who participated in the baseline survey intending to reside in a cluster in the study area for at least next 12 months are eligible for the trial. Further, participants will be included in the trial if they are diagnosed cases of type 2 diabetes with fasting blood glucose ≥ 7.0 mmol/L and if they provide informed consent to participate according to the Declaration of Helsinki. Any participants who are severely ill, who are unlikely to be in the community throughout the intervention, pregnant women, and those who decline consent will be excluded from the study. Participants not included will be marked, meaning that a flowchart of numbers of invited, included, and drop outs will be produced.

### Trial interventions

We reviewed some of the available diabetes management guidelines from the WHO [25], the American Diabetes Association [26], and the International Diabetes Federation [27]. However, these guidelines were not entirely appropriate for the local population and regional needs. Moreover, Nepal does not have a national guideline for diabetes management care and, therefore, service providers use a range of guidelines [8]. Considering these facts, we aimed to develop a local guideline for the management of diabetes, based upon available evidence from the guidelines mentioned above. In this regard, a panel consisting of clinicians, health education experts, health workers, trained nurses, dieticians, and diabetes patients will be formed to develop a guideline appropriate for the local context and that considers the most reliable scientific evidence to determine management approaches in dealing with diabetic patients, especially with regard to priorities and effectiveness in the prevention and control of diabetes and its complications.

### FCHV intervention (training component)

We will train 20 FCHVs of the intervention cluster for diabetes management. The FCHVs will receive a 5-day training package highlighting (1) an introduction to NCDs/diabetes; (2) identification of diabetes risk factors using a checklist comprising physical inactivity, family history, smoking, and alcohol consumption; (3) screening techniques of blood glucose level, body mass index (BMI), blood pressure and other cardiovascular diseases, as well as referral for those who have poor glycemic scores; (4) providing health education (type 2 diabetes, cardiovascular diseases, their risk factors, meal planning, exercise training activity, maintaining or reducing body weight, smoking cessation, and personal hygiene); (5) importance of regular use of medication; and (6) recording, reporting and follow-up. An overview of program content is presented in Table 1. The training materials will be reviewed and validated by educational experts and stakeholders. The FCHVs will also receive health education materials (poster, pamphlet), test strips, glucometers, tape, digital sphygmomanometer, and a recording register during training sessions. The developed materials will be pretested with the FCHVs from a nearby area not included in the study. At the end of training, FCHVs will sit together and assign the number of households that have to be visited based on the baseline survey. Consequently, FCHVs will visit each household, meet the selected participant, and ask for informed consent to participate in the trial.

**Table 1:** Content of diabetes management training for female community health volunteers (FCHVs)

Day	Content
Day 1	Introduction of COBIN-D study
	Type 2 diabetes and its situation in Nepal
	Theoretical aspects of diabetes
Day 2	Diabetes classification and its major risk factors
	Measurement of blood glucose, blood pressure, height, and weight
Day 3	Counseling and health promotion messages on major risk factors and medication compliance
	Practical session for counseling
Day 4	FCHV visits
	Recording and reporting
Day 5	Selection of households
	Evaluation and certification

### Providing intervention to the participants at household level

Participants in the intervention clusters will receive a 12-month, family-based, home health education package administered by FCHVs in a household setting. On average, one FCHV will intervene six (range, 2–8) diabetic patients three times a year. During the visit, FCHVs will screen blood glucose and blood pressure, measure BMI, and deliver the health education intervention, focusing on increasing physical activity, reducing alcohol consumption, avoiding smoking, and nutrition education as well as discussion on diabetes and its complications. For example, during counseling, FCHVs will explain the prevalence of type 2 diabetes and risk factors. These discussions are intended to make participants aware of their risk and to enhance acceptance of their diabetes diagnosis. Educational sessions will be guided by the Health Belief Model [28] and Social Support Theory [29]. It is argued that adaptation of the Health Belief Model conceptualization of behavioral intentions contributes to a better understanding of the relationship between health beliefs and behaviors at the community level. Moreover, social networking and support might take the form of messages showing empathy, encouragement and caring (among others), which may be advantageous for health and positive mental attitude, including motivation for behavior change. If, during the visit, participants have a fasting blood glucose level ≥ 7.0 mmol/L, they will be referred to the nearest health facility. Additionally, those on antidiabetic medication will be followed up for adherence to their medication during the FCHV visit. Each visit will provide participants with the opportunity to discuss individualized care needs related to diabetes management with the FCHV, as well as to discuss goal-setting around health behaviors using motivational interviewing techniques. The FCHV activities will be cross-verified by the field supervisor and will be confirmed during the follow-up survey by asking participants whether FCHVs had adhered to the protocol. We will also use a supervision checklist to track and update the knowledge and skill levels of FCHVs as well as to ensure fidelity to the study protocol.

In the wait-list control clusters, participants will receive ‘usual care’ by continuing to manage their glycemic condition as usual. They will not receive further contact, information, or educational materials from FCHVs until the 12-month assessment. They will be invited to receive the intervention after completion of the 12-month assessment. Immediately after the follow-up survey, we will deliver health education intervention for wait-list control groups by conducting a 3-day workshop where participants will be provided health education on diabetes management.

### Trial outcomes

Outcome measures will be collected from participants in both intervention and control clusters at baseline and after 1 year of intervention.

### Primary outcome

The primary outcome measure of the study will be the difference in mean change (from baseline to 1 year) in fasting blood glucose between the two study arms. Fasting blood glucose is likely to be the most feasible measurement in LMICs [30].

### Secondary outcomes

Differences in risk factors, including smoking history, alcohol use, daily servings of fruits and vegetables, BMI, obesity, physical activity, blood pressure, HbA1c and medication compliance between the two study arms.

### Sample size calculation

The sample size for the baseline survey will be calculated with 95% confidence interval (z = 1.96), based on the following assumptions: margin of error (alpha), 5%; estimated prevalence of diabetes, 9.5% [31]; design effect, 2; and anticipated response rate, 80%. We will only include the ≥ 25 years age group, so we will have four age groups for each sex (total strata = 8); this will result in a sample size of 2643. The sample size was calculated based on the method suggested by the WHO STEPWISE Approach [32]. According to the STEPS sample size calculator, a margin of error of 0.05 is suggested in a prevalence survey for an expected prevalence of 10% or greater. In addition, a smaller margin of error of 0.02 or 0.01 is considered appropriate when the expected prevalence is lower than 10%. The anticipated prevalence of type 2 diabetes was 9.5% in the earlier study [31], which is very close to 10%; this being an assumption, a margin of error of 0.05 was included. For the intervention study, we took a reference of a lifestyle intervention study for diabetes management conducted in a rural community-based setting, which showed a mean reduction in fasting blood glucose by 1.0 mmol/L after intervention in the diabetic population [33]. Using the two-sided *t* test with a significance level of 0.05 and assuming a standard deviation of 2.1, considering an intracluster correlation of 0.01, and the design effect of 1.1 and 80% power, we will need seven clusters with 13 individuals per intervention arm. Allowing for up to 20% loss to follow-up, the sample size was adjusted to 16 in each cluster, i.e., 112 individuals per arm or a total of 224 diabetes patients.

### Randomization

The units of randomization are clusters. We will include the clusters based on the one previously randomized by the COBIN study in a 1:1 ratio to the intervention (7 clusters; *n* = 112) and wait-list control (7 clusters; *n* = 112). Due to the nature of the intervention, it was not possible to blind subjects to their allocation.

### Data collection, management and analysis

#### Baseline and follow-up survey

The baseline survey will be performed in both the intervention and wait-list control clusters. A culturally adapted, Nepali-translated, and previously validated expanded version of WHO STEPwise Surveillance tool will be used to conduct the survey [34]. Data on demographics, behavioral risk factors, blood pressure, anthropometric measurements (weight, height, waist and hip circumference), and fasting blood glucose will be collected by face-to-face interviews during a door-to-door visit by eight specifically trained field workers with a background in health. However, blood lipid profiles, including cholesterol and triglycerides, will not be measured due to logistic limitations. Data collection and training will be performed in accordance with the WHO Stepwise approach recommended for NCD surveillance [35]. Participants will be asked to provide written consent to have fasting blood glucose (fasting being defined as no caloric intake for at least 8 hours) measured via a finger stick blood sample and glucometer (the Finetest Auto-coding™, Infopia Co., Ltd. Korea). On a pre-informed date, the fasting blood glucose test will be conducted in the morning. Participants will be requested to fast overnight (including no smoking or drinking tea in the morning) and will be reminded by telephone the day before the test. Fasting will be confirmed verbally by the participants immediately before collecting the blood sample. Blood pressure will be measured in the resting state as the average of three readings taken 5 min apart by using an Omron Automatic Blood Pressure Monitor. The Principal Investigator (PI) and a field supervisor will supervise data collectors. To ensure standardization and quality of the interview techniques and clinical measurements, including blood glucose and blood pressure, field workers will be trained for 5 consecutive days. The glucometers, sphygmomanometers, weighing scales, and tape measures will be calibrated weekly by taking measurements of one person on each of the instruments. Completed questionnaires will be validated in telephone interviews with selected participants. Households will be visited one or more times as needed in order to complete the questionnaires. The knowledge and skills of FCHVs will also be assessed by conducting a mixed-method study, including both qualitative and quantitative studies. The same methodology will be used to conduct a follow-up survey after 1 year in both intervention and wait-list control clusters.

#### Data management

To ensure data quality during all study phases, all assessments and data forms will be checked on the day of completion by the PI. Only the PI will be allowed to correct erroneous information. Data will be coded and entered as the study progresses. The hard copy questionnaire will be stored in the field office accessible by the PI only. Edit checks will be performed to verify data as needed and will be entered in an EpiData software file (EpiData Association, Odense, Denmark). Electronic data will be stored in the EpiData software.

### Statistical analysis

Quantitative data analysis will be performed using STATA version 14.1 software (StataCorp, College Station, TX, USA). The primary analysis will be conducted under the principal of intention-to-treat, i.e., without regard to the compliance of individuals within their allocated study arm and with clustering effects. All dropouts will be accounted for and reported. Reasons for dropouts will be mentioned. A second analysis that will impute missing data will be used for handling missing data. For individual outcomes, proportions will be compared using χ^2^ test and continuous measures will be compared using *t* tests. Random effect mixed regression analysis will be adjusted for age and sex. Since randomization will be at the cluster level in the study, a random effects model will be used to account for clustering effects. The effectiveness of the intervention will be tested by analysis of covariance, which will allow us to adjust for baseline differences between groups. Two-sided *p* values will be reported at 95% confidence interval with a statistical significance level of less than 0.05.

### Quality control procedures

Quality controls are conducted during all study phases. We will conduct a week-long training workshop on data collection techniques of the WHO STEPwise Surveillance tool for field workers. Training will include lectures on the study objectives, STEPS survey questionnaires, interviewing techniques, and mock interviews between enumerators to practice interviewing. The field workers will pre-test the questionnaire in the presence of the field supervisor on a sample population from a nearby village not included in the study. Furthermore, we will train field workers in both the theoretical and practical aspects, including the use of the glucose measuring device and instructions of quality control. Further, we will calibrate the glucose monitors to properly recognize the test strips currently in use. We will also use pre-defined blood glucose control cut-off points for fasting blood glucose as recommended by the WHO and report cases lost to follow-up in the follow-up survey. Additionally, during the intervention phase, the developed training materials will be pretested with the FCHV from a nearby area not included in the study. The field supervisor will undertake monthly visits to all households from the intervention clusters, record their observations using an observation checklist regarding intervention delivery, protocol adherence, and adverse event recording and reporting. Regular monitoring will be performed by the field supervisor to identify challenges faced in the intervention and to address issues and gaps identified. The supervisor and FCHVs will meet every month to review and discuss results and their implications. Quarterly booster sessions will be conducted to review home-based counseling skills and blood glucose screening techniques for FCHVs.

### Data integrity

Data integrity will be enhanced by having data collected by a number of trained field workers with a healthcare background and by adherence to assessment protocols.

### Minimization of contamination

All possible actions will be taken to reduce and minimize contamination in this study. Including the same intervention and wait-list control clusters as previously reported in the COBIN study will presumably help to provide larger intervention effects as well as to avoid contamination. Further, our clusters are sufficiently distant and well separated from each other, which may reduce bias.

### Cost and compensation

FCHVs will be reimbursed for transport costs and refreshments (US$5) for each visit. The study carries very minimal risk to the study participants, which is why we have no compensation or insurance plan for the participants.

### Dissemination of results

The results will be disseminated through workshops, local and international conferences, meetings and events organized with local stakeholders, and in peer-reviewed publications.

## Discussion

The COBIN-D is the first study investigating the effect of family-based health education and screening on blood sugar levels in adults by FCHVs at the community level in Nepal. The perspective of this study is to develop and implement, in collaboration with the community people, a community-based, culturally sensitive diabetes prevention and control program. Monitoring blood glucose with point-of-care testing at home can diagnose as well as monitor how well diabetes is controlled. At the same time, the study is highly interdisciplinary and combines culturally appropriate health education and health promotion approaches to diabetes education, engages and trains locally available FCHVs for NCD prevention including diabetes, and establishes a local coalition whose focus is on increasing awareness and promoting healthy lifestyle changes. Involving local volunteers will help address social, cultural, political, and economic systems to change the health behaviors and outcomes of the community, which ultimately supports the high priority given to research translation into new practices and policies. The assessment of FCHV skills may help to develop a policy that can be scaled-up to the national level. If the health promotion package is found to be effective, the approach can be easily adopted into the existing healthcare delivery system in Nepal. The study output can bring immediate benefits to the individuals of the intervention area as well as contributing to the development of a national diabetes management plan. It is anticipated that the study can act as a feasible and affordable tool for evidence-based integrated care for improvement of diabetes management and outcomes in Nepal as well as in LMICs elsewhere.

Some of the decisions taken in this protocol require clarification. The fasting blood sugar level will be measured only once in our study; we are aware that one measurement is not precise, yet this is not being implemented for the provision of medication. We recognize that calculating the mean of two measurements is more precise, yet we were not able to implement this procedure. Therefore, a false positive rate of approximately 20% is expected. However, we will use the same definition for both intervention and control, which will neutralize this effect. There is evidence that a single blood glucose measurement is an alternative to HbA1c or oral glucose tolerance test for community surveillance [36]. Additionally, there may also be contamination issues. All possible actions will be taken to reduce and minimize contamination in this study. Interventions will be allocated at the cluster rather than the individual level to minimize contamination among participants within the same cluster. Our FCHVs will be informed not to share information about the study and not to provide any support to people from other clusters in the community, other than those which they have been assigned for intervention during the training. We have plans to collect the information related to possible contamination during the follow-up survey. We will consider any contamination during the outcome estimation and adjust this while estimating the effect.

### Trial status

**Fig. 2:**
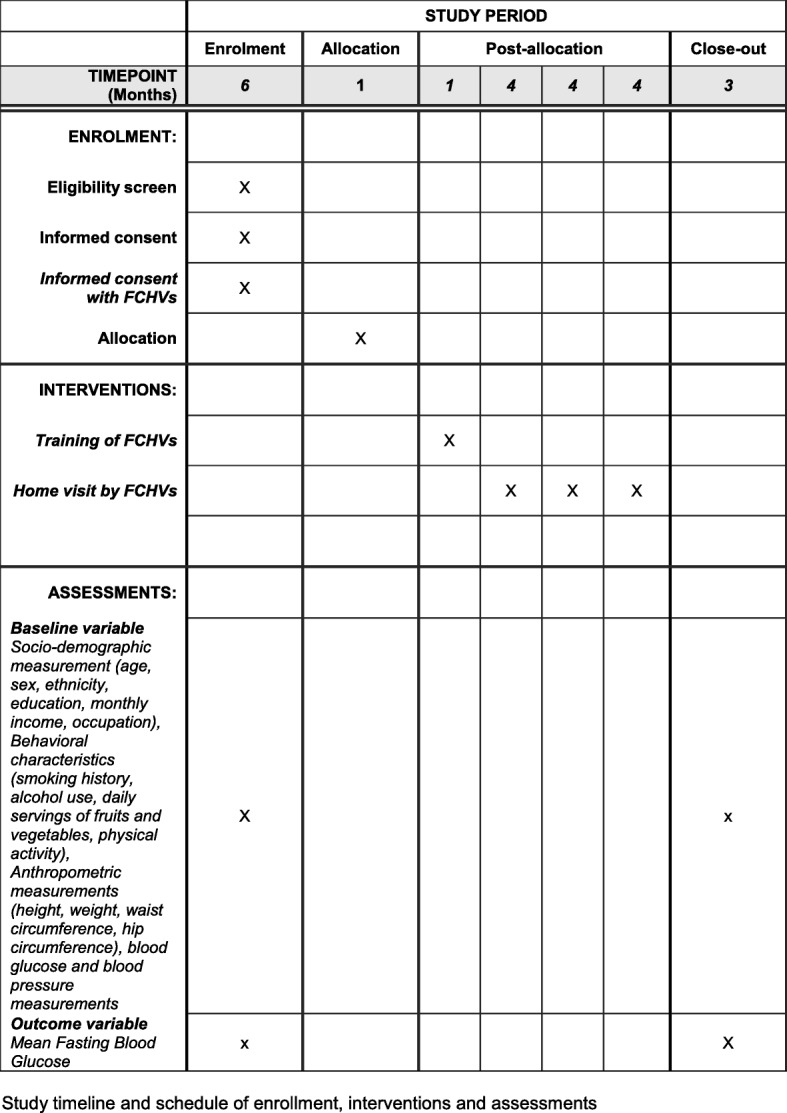
Schedule of enrollment, interventions, and assessments

The trial is now closed to participant accrual, but the trial is ongoing. Participant recruitment of this trial began on June 2017. The endpoint assessment of all the participants will be completed by the end of October 2018. The schedule of enrollment, interventions, and assessments is presented in Fig. 2.

## Additional file

Additional file 1: SPIRIT 2013 Checklist: Community-based intervention for management of diabetes in Nepal (COBIN-D trial): study protocol for a cluster-randomized controlled trial. (DOC 121 kb)

## Abbreviations

BMI: body mass index
cRCT: cluster randomized controlled trial
COBIN: Community Based Management of Non Communicable Diseases in Nepal
COBIN-D: COmmunity Based Intervention for Management of Diabetes in Nepal
FCHVs: female community health volunteers
LMICs: low- and middle-income countries
NCDs: non-communicable diseases
PI: principal investigator
WHO: World Health Organization
